# Behavioral studies and veterinary management of orangutans at Bukit Merah Orang Utan Island, Perak, Malaysia

**DOI:** 10.1007/s10329-018-0650-2

**Published:** 2018-01-30

**Authors:** Misato Hayashi, Fumito Kawakami, Rosimah Roslan, Nurhafizie M. Hapiszudin, Sabapathy Dharmalingam

**Affiliations:** 1 Primate Research Institute, Kyoto University, 41-2 Kanrin, Inuyama, Aichi 484-8506 Japan; 2 Institute for Advanced Study, Kyoto University, Kyoto, Japan; 3 Bukit Merah Orang Utan Island Foundation, Semanggol, Perak Malaysia

**Keywords:** Behavioral monitoring, Ex situ conservation, Orangutan, Veterinary management

## Abstract

The Bukit Merah Orang Utan Island (OUI) Foundation has been conducting behavioral and veterinary research on orangutans as an attempt at ex situ conservation. Since 2010, the Primate Research Institute, Kyoto University has been collaborating with OUI to promote environmental enrichment and infant rearing by biological mothers in addition to the continuous efforts of refining the veterinary management of the endangered species. In 2011, three Bornean orangutans (*Pongo pygmaeus pygmaeus*) were released on an island, called BJ Island, adjacent to OUI. This island is approximately 5.6 ha in size, and 635 trees belonging to 102 plant species were identified prior to their release. Behavioral monitoring of the released individuals has been conducted to evaluate their behavioral adaptation to the new environment. Two of the three released orangutans were born in the wild, whereas the youngest individual was born on OUI and expected to learn forest survival strategies from the two older individuals. One of the orangutans was pregnant at the time of release and subsequently gave birth to two male infants on BJ Island. The behavioral monitoring indicated that these orangutans traveled more and spent more time on trees following their release onto BJ Island. However, resting was longer for two females both on OUI and BJ Island when compared to other populations. The orangutans consumed some natural food resources on BJ Island. The release into a more naturalistic environment may help the orangutans to develop more naturalistic behavioral patterns that resemble their wild counterparts.

**Electronic supplementary material:**

The online version of this article (10.1007/s10329-018-0650-2) contains supplementary material, which is available to authorized users.

## Introduction

Orangutans are the only species of great ape found in only two Asian countries, namely Malaysia and Indonesia (Delgado and van Schaik 2000; Husson et al. 2009). In 2016, the Bornean orangutan (*Pongo pygmaeus*) was categorized as a critically endangered species in the International Union for Conservation of Nature Red List (Ancrenaz et al. 2016). To cease the decline in the number of orangutans, effective in situ and ex situ conservation and management techniques need to be developed and implemented through international collaborations. Compared to the African great apes that live in social groups comprising multiple adult individuals, orangutans have a relatively solitary lifestyle, with only mothers and their infants remaining in constant physical proximity and contact, although the existence of loose social organization and regional variation has been suggested (Russon 2009a; van Schaik et al. 2009). Considerable number of orangutan rehabilitation and re-introduction efforts has been reported from 12 projects (eight of which were active in 2009: six in Borneo and two in Sumatra), and effective protocols, as well as practical constraints have been suggested in these sites ranging within orangutans’ natural habitat (Russon 2002, 2009b; Grundmann 2006; Descovich et al. 2011; Kuze et al. 2008, 2012; Robins et al. 2013).

In Malaysia, part of Borneo is home to two subspecies of orangutans: *P. pygmaeus pygmaeus* and *P. pygmaeus morio*. Wild orangutans have already disappeared from Peninsular Malaysia, but fossil records of orangutans have been found there (Tshen 2016). Ibrahim et al. (2013) reported that orangutans persisted in Peninsular Malaysia around 33–66 thousand years ago, but declined in number because of climate change and paleogeographical constraints that prevented the reconstruction of local populations even after the climatic amelioration in the Late Pleistocene.

Orang Utan Island (OUI), which is located in Perak, Peninsular Malaysia, was established in February 2000 with assistance from the Bukit Merah Laketown Project and EMKAY Group (http://emkay.com.my/). The early operations are reported in Agoramoorthy (2002). OUI currently houses 22 orangutan individuals, the family lineage of which is shown in Table 1. Eleven orangutans (*P. pygmaeus*) were initially introduced from Sarawak Forestry and Malacca Zoo, Malaysia, between 2000 and 2002, four of which have died. Since then, 21 orangutans have been born on OUI and successfully lived there for more than 2 years, indicating the reproductive success of this endangered species, which is considered rather difficult to breed in captive settings of some countries without a strategic breeding program such as the Species Survival Plan operated in North America region (Cocks 2007). Initially on OUI, if an infant had health problems, it was separated from its mother to provide intensive veterinary treatments and care by humans for 24 h per day. This practice enabled the accumulation of veterinary knowledge on orangutan development, as well as protocols for veterinary care and managements, behavioral rehabilitation, and introduction to an enclosure with other conspecifics (Dharmalingam et al. 2012; Sabapathy 2013).

**Table 1:**
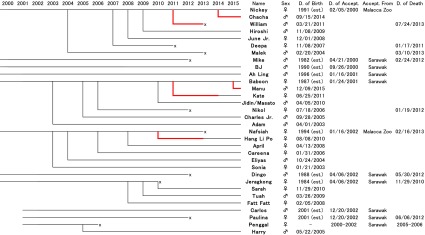
Family lineage of Orang Utan Island (OUI) orangutans. Red line shows the period of mother-rearing

In 2008, the Bukit Merah Orang Utan Island Foundation was established, which aimed to promote an effort for the ex situ conservation of orangutan’s population. OUI receives many visitors (42,747 in 2015), school visits for education programs (2,823 students in 2015), researchers, and internship students (such as from Universiti Sains Malaysia as reported by Mashhor and Anuar 2013). Thus, OUI functions as a window for environmental education on the peninsular side of Malaysia, and attracts both local and international visitors.

In 2010, the Primate Research Institute, Kyoto University, started collaboration with OUI. We shifted the key focus to the promotion of environmental enrichment and infant rearing by the biological mother, two essential standards for keeping great apes (McCann et al. 2007). Mother rearing has been shown to be particularly important for infants’ cognitive and social development in great apes (Hayashi and Matsuzawa 2017). African great apes live in social groups, and so their offspring are able to stay near to the mother in the natal group for at least several years after weaning. Orangutan offspring have the longest dependent period of approximately 7–8 years (van Noordwijk and van Schaik 2005); however, they need to be independent from the mother at the time of weaning because she may have difficulty accompanying two dependent offsprings at a time (van Adrichem et al. 2006; Mendonça et al. 2017). Since orangutans live in an environment where the amount of natural food resources always fluctuates (Kanamori et al. 2010, 2017), long-term postnatal learning from the mother about the site-specific environment and its variation through time is essential for the infant’s survival in the solitary life after weaning.

In 2011, three orangutans were released on BJ Island, which is adjacent to OUI, as the final stage of the behavioral rehabilitation program at OUI and the first step toward realizing the final goal of wild release. The rescued infant orangutans are supposed to undergo a rehabilitation program in a rehabilitation center located in their natural habitat, allowing them to be gradually habituated to forest living from infancy. In contrast, we released three orangutan individuals from juvenile and adult age categories. The present paper reports on the current situation of behavioral studies and veterinary management on OUI together with the results of behavioral monitoring of the ex-captive orangutans in an island of Peninsular Malaysia.

## Methods

### Subjects

OUI currently holds 22 orangutan individuals (Table 1). Three individuals kept on OUI were selected to be released on BJ Island on February 15, 2011. This included a subadult male Ah Ling, 14 years old at the time of release, an adult female Nickey, 19 years old, and a juvenile female Sonia, 8 years old.

The adult female Nickey was pregnant at the time of release and subsequently gave birth to a male offspring, William, in March 2011. Unfortunately, William died from respiratory illness in 2013. Nickey also gave birth to a second male offspring, Chacha, on BJ Island in September 2014.

### Facilities

**Fig. 1:**
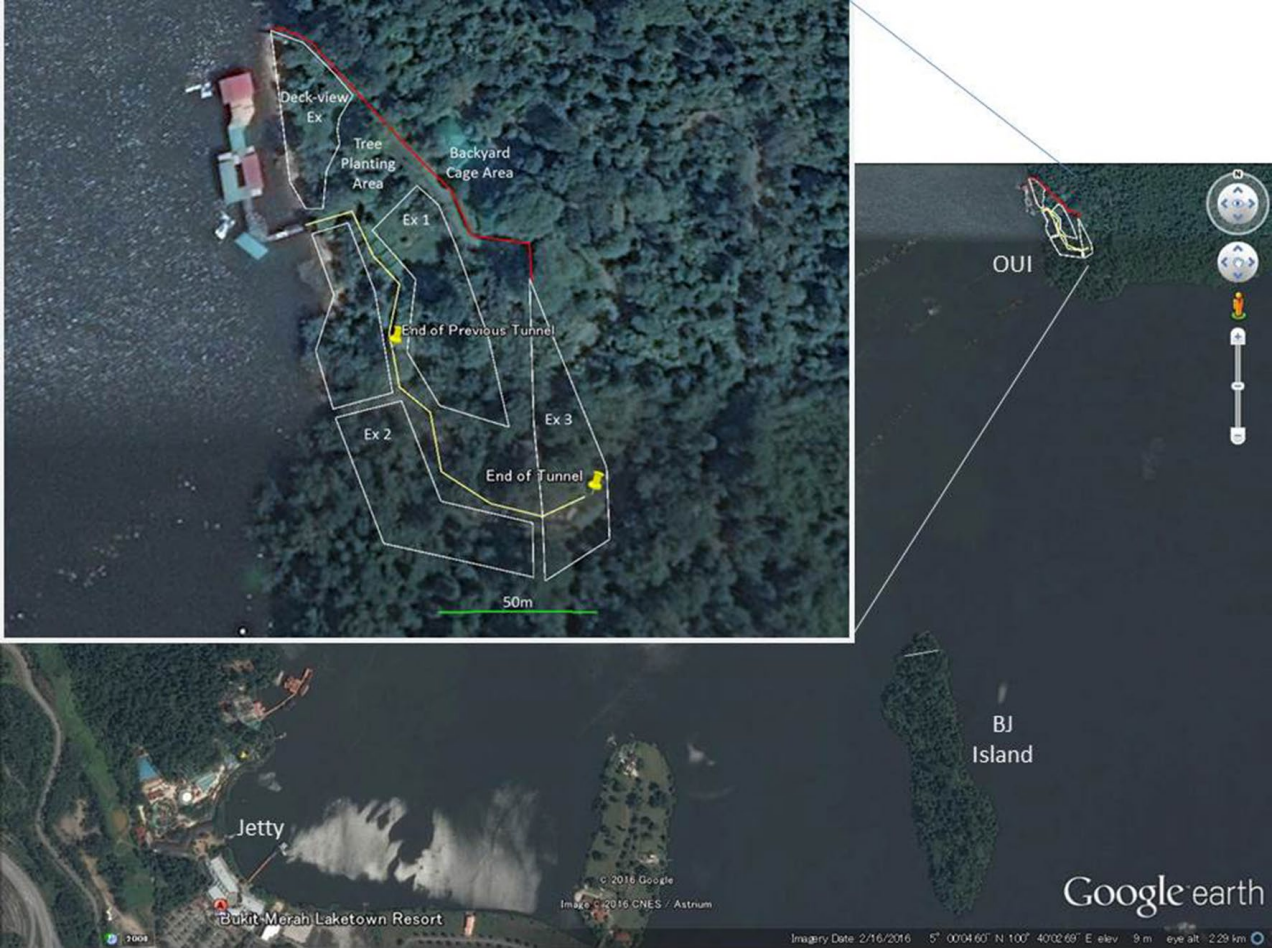
Overview of Orang Utan Island (OUI) and BJ Island

OUI currently operates five outdoor enclosures ranging from around 1000 to 2400 m^2^ that are separated by electronic hot-wire fences (Fig. 1). Each enclosure is covered by natural vegetation, and contains ropes connecting the trees, wooden platforms, and feeding devices. The orangutans have daily access to the outdoor enclosures from 9 am to 5 pm. During the night, they return to their night cages, where they are kept individually or with some cage mates.

Human visitors to OUI walk through the tunnel that is located in the middle of the orangutan enclosures, from which they can observe the orangutans moving freely in the enclosures through metal frames covering the tunnel. At the beginning of the collaboration with the Primate Research Institute, Kyoto University, OUI connected high trees in the orangutan enclosures by increasing the number of ropes to facilitate arboreal transfer between trees. In addition, some materials were attached to the cages in the holding area for feeding enrichment. The outdoor enclosures and the visitors’ tunnel were expanded in 2012, with the latter currently being approximately 200 m long, enabling the observation of orangutans in the enlarged outdoor-enclosures that contain a lot of natural vegetation and trees.

From 2010, mothers were encouraged to take care of their own infants for as long as possible. Most of the mothers had no difficulty holding their infants from the beginning and the skill of smooth breast feeding developed gradually through mother–infant interactions. In the case of orangutan mothers who had a good relationship with human caretakers, humans were able to give medication to the infants, and control both the mothers and infants in a direct keeping-style.

Only the infants who lost their mothers have been hand-reared by humans since 2010. Prior to this, the orangutan infants were closely cared for by humans at the Infant Care Unit (ICU) for 24 h per day until they were approximately 1 year of age. Infants of around 1–4 years of age were then kept as a group in the Enrichment Development Unit cage, which was provided with enrichment materials such as ropes, hammocks, and natural tree branches in order to develop their locomotor skills, during which time they spontaneously developed the social skills to interact and play with other individuals which are kept in the same group. After this stage, they were transferred to an outdoor enclosure to develop tree climbing skills, where they maintained their social interactions with conspecifics including adolescent and adult individuals.

During the final phase of hand rearing by humans, some behavioral studies were also conducted. The first author went into the same cage as the infant orangutans to conduct cognitive developmental tests that were originally developed to test and compare African great apes and human infants (Hayashi and Matsuzawa 2003; Hayashi 2007). Currently, no infants are kept in ICU and the Enrichment Development Unit, indicating the fundamental success of the rearing attempts by OUI orangutan mothers.

BJ Island is 1.1 km away from OUI and approximately 5.6 ha in size. Researchers and students from Pulau Banding Rainforest Research Centre, Universiti Sains Malaysia (USM), and OUI staff surveyed the vegetation on BJ Island before releasing the orangutans and identified 635 trees belonging to 102 plant species of 70 genera, 35 families (Supplementary Table). Prior to the release, we created a small human visiting area on the island that included a jetty, researchers’ hut, generator for the hot-wire fence dividing the two areas, and orangutan cage for quarantine purposes.

### Data collection and statistics

**Fig. 2:**
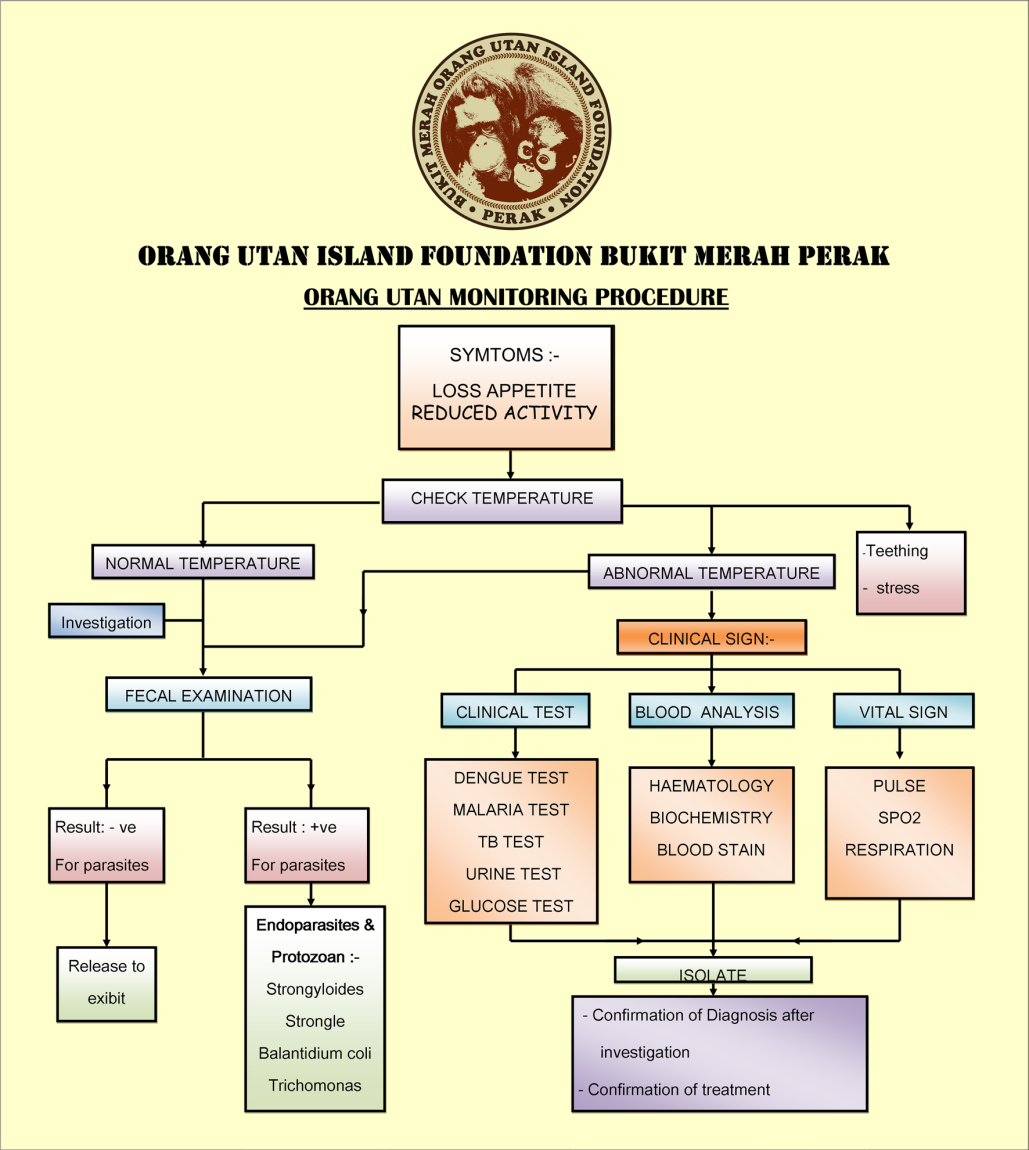
Flowchart of health monitoring procedure for orangutans in Orang Utan Island (OUI)

Health conditions of orangutans on OUI are closely monitored by veterinary staff. The procedure of health monitoring for orangutans, as well as the protocol for diagnosis and treatment has been established on OUI (Fig. 2). Previous studies have reported the veterinary management of orangutans in the captive setting of OUI (Dharmalingam 2015a, b, 2016).

Three orangutans from OUI were anesthetized, individually placed in transfer cages, and transferred to BJ Island, where they were released on February 15, 2011. Behavioral monitoring was conducted before and after the release of these orangutans using focal animal sampling at 1-min intervals (Martin and Bateson 2007). The present analysis used the behavioral data collected by local staff before (December 2010 and January 2011) and after (June, August, and September 2011) the release. Supplemental feeding has been conducted twice per day to compensate for the scarcity of natural fruiting trees on BJ Island. Minimal human contact has been maintained with the orangutans on BJ Island, with only the core keepers and research staff entering the orangutan area to feed and observe them.

We applied Pearson’s Chi-squared test for each individual by using R version 3.4.2 (2017 The R Foundation for Statistical Computing) to compare orangutans’ behavior on OUI and BJ Island. In order to apply the Chi-square test, five infrequent behavioral-categories (Social/Object Play, Social/Self Groom, and Others) were pooled into one category of “Miscellaneous”, although the percentage of each behavioral category is shown in Table 3. Exact number of recorded data-points in four behavioral categories (Feed, Rest, Travel, and Miscellaneous) were summed up in two sites (OUI and BJ Island) for each individual and used for the analysis. When the results were statistically significant, a residual analysis was conducted to detect, which behavioral category yielded the significant difference among the two sites (*p* < 0.05) by using a R-program provided by js-STAR. Exact number of recorded data-points for the stay on ground or arboreal were summed up in two sites for each individual and used for a Pearson’s Chi-squared test with Yates’ continuity corrections.

## Results

Orangutans sometimes developed symptoms and received veterinary care and treatment on OUI. Common diseases occurring on OUI are listed in Table 2. Top two diseases of Strongyloidiasis and Balantadidiasis were also common in other captive/rehabilitation centers and wild sites (Labes et al. 2010, Foitová et al. 2009). Amoebic dysentery caused by *Entamoeba* sp. and Ascariasis were less prevalent, but within the repertoire in seven and eight out of 10 populations, respectively. Although we lack comparable data for some diseases caused by bacteria, some diseases may be caused by the close proximity among conspecifics and with human visitors in the captive setting.

**Table 2:** Common diseases occur in Orang Utan Island (OUI). The frequency of occurrence was shown as the percentage of orangutan individuals infected by each disease per year. The right three columns show the comparable data on parasite infection from two previous studies

Disease	Pathogen	Transmission	Infected orangutans at OUI (% of individual per year)	Labes et al. (2010) Centres (% among identified parasites)	Labes et al. (2010) Sites (% among identified parasites)	Foitová et al. (2009) (number of infected places among 10 populations)
Strongyloidiasis	Nematode	Direct contact with contaminated feces	25	38	33	9/10
Balantadidiasis	Parasite (Protozoa)	Direct contact with contaminated feces. Animal-to-animal transmission	20	40	41	9/10
Amoebic dysentery^a^	Parasite (Protozoa)	Food, water, fomites, insects. Fecal–oral route	15	13	16	7/10
Melioidosis	Bacteria	Transmitted by inhaling dust contaminated by the bacteria and when contaminated soil comes in contact with abraded skin	10	–	–	–
Salmonellosis	Bacteria	Direct contact with infected animal. Ingestion of contaminated food or water	10	–	–	–
Ascariasis (roundworm)	Nematode	Ingestion of contaminated food or water. Animal-to-animal transmission. Direct contact with infected animals	10	1	0	8/10
Giardiasis	Parasite (Protozoa)	Ingestion of contaminated food or water. Animal-to-animal transmission. Direct contact with infected animals	5	4	3	2/10
Respiratory tract infection	Bacteria	Direct contact with infected animal. Inhalation of aerosol droplets	5	–	–	–

^a^Right three columns correspond to the results for *Entamoeba* sp.

**Fig. 3:**
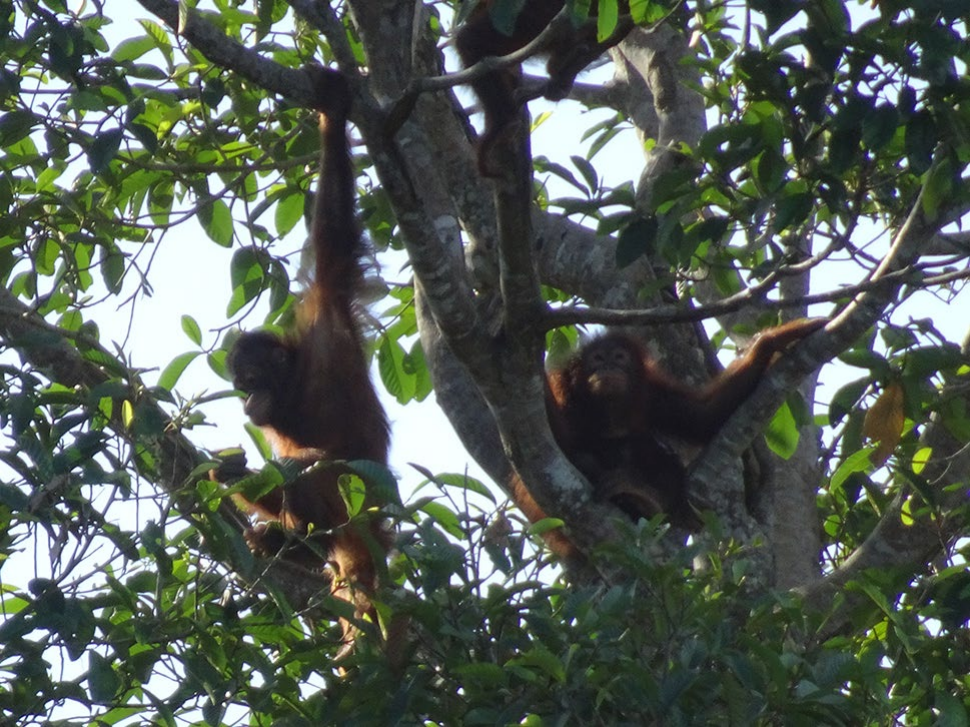
Orangutans in an outdoor enclosure in Orang Utan Island (OUI)

In the outdoor enclosures of OUI, the orangutans used the natural vegetation (Fig. 3) and ropes for locomotion, manipulative play, and making day nests. Play behavior was frequently observed between multiple individuals in peer groups of similarly aged young orangutans. Although the orangutans occasionally remained on the ground to play or interact with peers and human caretakers, they also climbed the trees for both playing and resting. Many interesting behavioral patterns that allow us to make inferences about the orangutans’ cognitive development were observed on OUI, including hitting or digging actions using an object in hand, the use of tools to touch and open the hot-wire fence, and social interactions with conspecifics and free-ranging crab-eating macaques (*Macaca fascicularis*).

A behavioral comparison of the three orangutans that were released on BJ Island with those on OUI revealed several patterns (Table 3). Activity budgets of three orangutans were significantly different between OUI and BJ Island (Ah Ling: *χ*^2^ = 120, *df* = 3, *p* < 0.0001, Nickey: *χ*^2^ = 20, *df* = 3, *p* = 0.0002, Sonia: *χ*^2^ = 150, *df* = 3, *p* < 0.0001). The residual analysis revealed significant differences (*p* < 0.05) as listed in below (Ah Ling: Rest and Travel was longer on BJ Island and Miscellaneous was longer on OUI, Nickey: Travel and Miscellaneous was longer on BJ Island and Rest was longer on OUI, Sonia: Feed and Travel was longer on BJ Island and Miscellaneous was longer on OUI). Thus, the consistent pattern among the three orangutans was prolonged traveling on BJ Island. Comparison to the previous report from four wild (three in Borneo and one in Sumatra) and four rehabilitant orangutans (three in Borneo and one in Sumatra); in total, eight populations (Russon 2009b) revealed that some behaviors were out of the range of activity budgets in previous reports. Resting was longer in two females both on OUI and BJ Island (62.1 and 54.7% for Nickey and 55.7 and 53.8% for Sonia) than the normal range in the wild (10.9–50.6%) and in rehabilitant (13.9–49.6%). On OUI, feeding for Sonia (10.4%) and traveling for Nickey (9.3%) were shorter than the normal range in the wild (31.9–69.3% for feeding and 12.2–18.2% for traveling) and in rehabilitant (20.8–64.1% for feeding and 10.8–18.7% for traveling). On BJ Island, traveling for Ah Ling (20.5%) and Sonia (21.0%) was slightly above the normal range in the wild (12.2–18.2%) and in rehabilitant (10.8–18.7%).

**Table 3:** Activity budgets for basic behavioral patterns in each orangutan on Orang Utan Island (OUI) and BJ Island

Name	Ah Ling		Nickey		Sonia		Russon (2009b)	
Place	OUI	BJ	OUI	BJ	OUI	BJ	Wild	Rehabilitant
Observation bouts (total length in min)	3 (212)	6 (463)	6 (483)	14 (1381)	7 (492)	14 (1365)		
% Feed	24.5	24.8	25.5	23.8	10.4	21.2	31.9–69.3	20.8–64.1
% Rest	20.8	47.5	62.1	54.7	55.7	53.8	10.9–50.6	13.9–49.6
% Travel	14.2	20.5	9.3	15.0	13.6	21.0	12.2–18.2	10.8–18.7
% Social play	15.6	1.7	0.0	6.2	4.1	1.3		
% Object play	1.4	0.0	0.4	0.0	4.7	1.0		
% Social groom	19.8	1.9	0.0	0.0	0.0	0.1		
% Self groom	2.4	1.9	0.0	0.0	0.6	0.4		
% Others	1.4	1.5	2.7	0.4	11.0	1.1	0.0–3.3	0.0–11.6
% Ground	60.8	22.2	84.9	48.7	61.0	41.4		
% Arboreal	39.2	77.8	15.1	51.3	39.0	58.6		

The right two columns show the data range reported from four wild and four rehabilitant orangutans reported in Russon (2009b). Underlined value was below (single-line) or above (double-line) the previous data-range from wild and rehabilitant orangutans reported in Russon (2009b)

Positional behavior of three orangutans (stay on ground vs arboreal stay) were significantly different between OUI and BJ Island (Ah Ling: *χ*^2^ = 94, *df* = 1, *p* < 0.0001, Nickey: *χ*^2^ = 190, *df* = 1, *p* < 0.0001, Sonia: *χ*^2^ = 55, *df* = 1, *p* < 0.0001). All subjects stayed longer on trees rather than on ground on BJ Island.

**Fig. 4:**
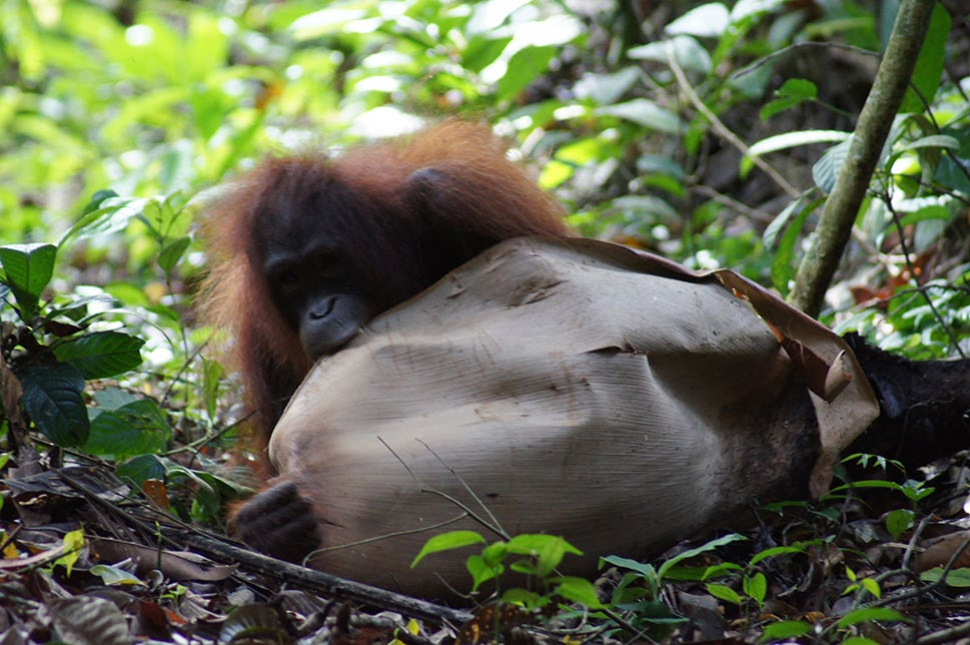
The youngest orangutan on BJ Island (Sonia) playing with a large fallen leaf

The orangutans that were transferred to BJ Island successfully made a nest from the first night. They also occasionally consumed natural vegetation on the island (see the detail in Supplementary Table) in addition to the supplemental fruits and vegetables provided by humans. Orangutans consumed 14 species (14 genera of 10 families) from natural vegetation on BJ Island: fruits of two species, leaves of 11 species, bark of three species, stems of one species, and shoots of one species. The youngest individual, Sonia, consumed more food items (13 items) compared to older individuals (Ah Ling: seven items, Nickey: five items). However, comparison with previous reports from 11 wild (eight in Borneo and three in Sumatra) and four rehabilitant (three in Borneo and one in Sumatra) populations (Galdikas 1988; Kanamori et al. 2010; Russon et al. 2007; Russon et al. 2009) showed that only 10 species were consumed by orangutans of BJ Island, as well as the other sites. Four species were consumed only by orangutans of BJ Island and only leaves and stems were consumed in this category. Although 78 species were present on BJ Island and were recorded as food items in other sites, orangutans on BJ Island did not consumed these plant species. Remaining 10 species were present on BJ Island, but orangutans on BJ Island nor other sites do not consume these plants. The orangutans occasionally encountered and interacted with each other in the forest or near the human area. The youngest individual also used natural vegetation for object play (Fig. 4).

**Fig. 5:**
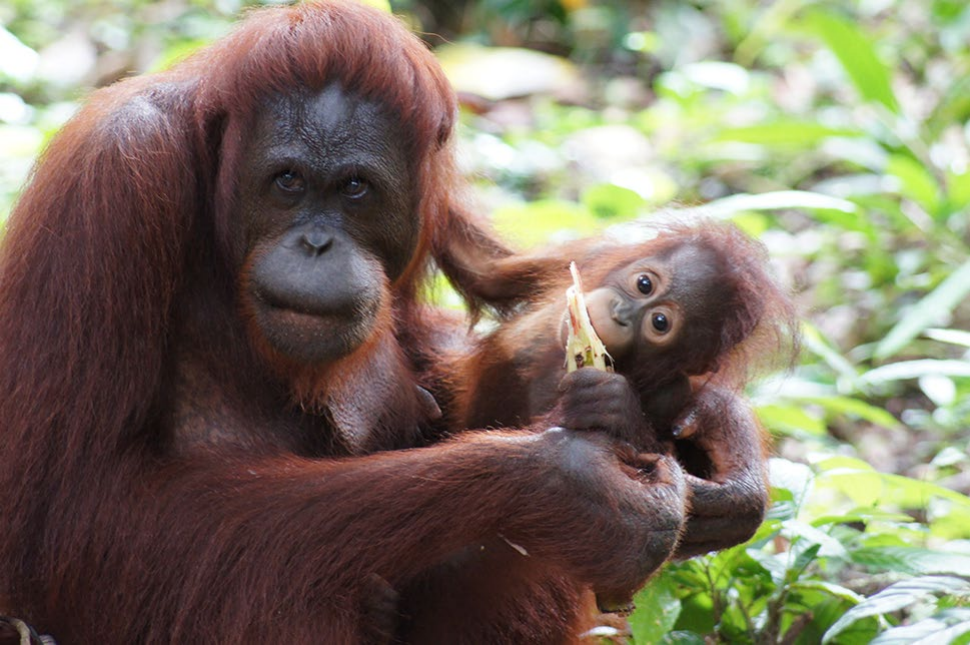
Infant orangutan (William, 1 year 6 months old) eating a sugarcane from the hand of his mother (Nickey)

The mother, Nickey, had four previous births before giving birth to her son, William, on BJ Island. She experienced initial difficulty in breast feeding the baby smoothly as she had no previous experience in taking care of her offspring for an extended period of time. For the first few weeks, the baby emitted whimpers when he needed breast feeding, but failed to contact the mother’s nipple on his own. Through these mother–infant interactions, the mother gradually learned appropriate maternal behaviors, including breast feeding. Intensive mother–infant interactions including play behaviors were observed throughout the study period and food sharing frequently occurred between the mother and infant after the soliciting behaviors from the infant (Emi Yamamoto, personal communication; Fig. 5). Although the first infant born on BJ Island (William) died from a respiratory disease, the mother succeeded in rearing her own offspring under the naturalistic conditions of the forest for more than 2 years. Moreover, she gave birth to her next offspring on BJ Island 1 year and 1 month after the death of William.

## Discussion

Activity budget of three orangutans were significantly different on OUI and BJ Island. More specifically, time spent for traveling and arboreal stay was significantly longer on BJ Island constantly across all three orangutans. In the subadult male, the social interaction with conspecifics was decreased after the release on BJ Island. These results may indicate that the more naturalistic environment yielded the emergence of more appropriate behavioral patterns which resemble to those of wild counterparts. In comparison to the range of activity budgets reported from other orangutan populations, resting time for two females was longer both on OUI and BJ Islands. Feeding of younger female and traveling of adult female was shorter on OUI compared to other populations. Time for traveling was slightly above the normal range for the male and younger female on BJ Island. These results may indicate that BJ Island was a more suitable environment for eliciting more naturalistic behavior in orangutans. However, even on BJ Island, the resting time of females was above the range in other populations, indicating that we need more effort in achieving the activity budget, which is closer to the wild orangutans (Morrogh-Bernard et al. 2009).

Unfortunately, it was necessary to provide the orangutans on BJ Island with supplemental foods due to the scarcity of fruiting trees here. Activity budget for feeding was less and resting was more than the normal range in some of the orangutans on BJ Island. This result can be explained by the provisioning of nutritious fruits and vegetables which decreased the necessity of foraging in the forest. Thus, the number of natural food items consumed by orangutans on BJ Island (14 species) was very low compared to the wild orangutans’ food repertoire (Leighton 1993) and we found only 10 species were consumed by both parties. Therefore, if we think about the next step of wild release, we need to further improve the foraging skills of the orangutans by increasing food repertoire to the species present in Peninsular Malaysia and within the food repertoire of wild orangutans (78 species on BJ Island).

As these orangutans were kept in human proximity for many years, it may be difficult to keep them at an appropriate distance from humans, making behavioral monitoring difficult as suggested by a previous study (Smith 2009). The present paper reports on a rare case of rehabilitation effort of individuals including adult, whereby the subadult male, Ah Ling, developed his cheek pad during the behavioral monitoring on BJ Island and became more aggressive toward humans. The youngest orangutan had no fear of artificial objects and so broke the trap camera after exploration. This sort of habituation is more typical and makes it difficult to monitor behavior using trap cameras.

OUI currently promotes mother rearing to support natural development in infant orangutans. Although some mothers on OUI and BJ Island had initial difficulties with breast feeding, they never rejected holding their infants. Younger individuals carefully observed mothers taking care of their infants and sometimes interacted with the infants. These hands-on experiences may lead to the development of normal maternal behavior in the young females who were born on OUI and were not reared by their biological mothers.

Although wild orangutans do not naturally occur in Peninsular Malaysia, this region was historically a part of their range, making it easier to conduct ex situ conservation. As a zoo environment, OUI can provide an ideal environment for orangutans in terms of ambient temperature, basic vegetation, and local fruits as they are very close to their wild habitat. In addition, visitors to OUI are able to see the orangutans climbing trees, and this direct experience may lead them to think about their natural habitat and orangutan conservation in the wild habitats. Thus, OUI is a suitable place for conducting environmental education, as well as behavioral and veterinary research on orangutans.

The common diseases occurred on OUI were also reported from other facilities and sites. The establishment and refining of standard protocol for treatment of common diseases in captive environment is one of the essential issues for promoting orangutan conservation. Although human contact was minimized on BJ Island, some problems occurred during the rehabilitation process. Since the orangutans had spent a long time in human proximity, it was hard to maintain an appropriate distance between humans and the orangutans during behavioral monitoring. This may explain the cause of death of the first infant born on BJ Island (William), who may have contracted a respiratory disease from a human observer. Similarly, Kuze et al. (2012) indicated that the risk of infant mortality is higher for rehabilitated orangutan mothers. However, it is important to continue these conservation and rehabilitation efforts for endangered orangutans. The careful establishment of an appropriate protocol for veterinary managements and behavioral rehabilitation of ex-captive orangutans adapting into semi-wild environments is needed, and these attempts should be continuously monitored and evaluated through behavioral observation.

## Electronic supplementary material

Below is the link to the electronic supplementary material.

Supplementary material 1 (DOCX 36 kb)
