# Supplementary material for: Behavioral studies and veterinary management of orangutans at Bukit Merah Orang Utan Island, Perak, Malaysia
Source: Primates. 2018 Jan 30;59(2):135–44. doi: 10.1007/s10329-018-0650-2 (PMC5843681; doi:10.1007/s10329-018-0650-2)
Supplement: Supplementary file 1 — Supplementary material 1 (DOCX 36 kb) [file 10329_2018_650_MOESM1_ESM.docx]

**Supplementary Table**

List of plant species found on BJ Island. Number of trees shows the count for trees which have more than 10 cm of DBH (Diameter at Breast Height) reported from the team census. Food parts consumed by the three orangutans were shown in the right three columns (F: fruit, L: leaf, S: stem, Sh: shoot, B: bark). The bottom two species were artificially planted on BJ Island before the release of orangutans. The right three columns show the data from wild orangutans reported in Kanamori et al. (2010), Russon et al. (2007, 2009), and Galdikas (1988). In this comparison, the food item reported in other sites were from the same genus but different species, we showed it in parenthesis such as (F).

| Family | Scientific Name | Local Name | Number of trees | Ah Ling | Nickey | Sonia | Kanamori et al. (2010) | Russon et al. (2007, **2009**) | Galdikas (1988) |
| --- | --- | --- | --- | --- | --- | --- | --- | --- | --- |
| Anisophylleaceae | *Anisophyllea corneri* | Delek | 8 |  |  |  | F | L |  |
| Annonaceae | *Cyathocalyx pruniferus* | Antoi | 3 |  |  |  |  | F |  |
| Annonaceae | *Monocarpia marginalis* | Mempisang | - |  | L | L |  | F |  |
| Annonaceae | *Polyalthia sp.* | Mempisang | 18 |  |  | Sh | F, L, B | F, L, B | F |
| Annonaceae | *Xylopia ferruginea* | Jangkang bukit | 3 |  |  |  |  | F, (L), B | (F), (L), (Fl) |
| Apocynaceae | *Dyera costulata* | Jelutong | 1 |  |  |  |  | B | (F), (B) |
| Araliaceae | *Arthrophyllum diversifolium* | Lupa dahan | 3 |  |  |  |  |  |  |
| Arecaceae | *Eugeissona tritis* | Bertam | - | L, S | S | L, S |  |  |  |
| Arecaceae | *Oncosperma horridum* | Bayas | 1 |  |  |  |  | F, S |  |
| Arecaceae | *Pinanga sp.* | Pinang | - |  | L |  |  | F, S |  |
| Burseraceae | *Santiria apiculata* | Kedondong | 5 |  |  |  | F, (B) |  | F, L, B |
| Burseraceae | *Santiria laevigata* | Kedondong | 2 |  |  |  |  |  | F, L, B |
| Clusiaceae | *Garcinia atroviridis* | Asam gelugur | 34 |  | F | B | F, B |  |  |
| Clusiaceae | *Garcinia griffithii* | Kandis gajah | 9 |  |  |  | F, B |  |  |
| Clusiaceae | *Garcinia parvifolia* | Kandis burung | 18 |  |  |  | F, B | **F, L** |  |
| Clusiaceae | *Garcinia penangiana* | Kandis | 1 |  |  |  | F, B |  |  |
| Clusiaceae | *Mesua ferrea* | Penaga lilin | 1 |  |  |  |  | (L), (B) |  |
| Clusiaceae | *Mesua lepidota* | Penaga | 1 |  |  |  |  | (L), (B) |  |
| Dilleniaceae | *Dillenia ovata* | Simpoh | 1 |  |  |  | (B), (Fl) | F, L, B, Fl | (F) |
| Dilleniaceae | *Dillenia reticulata* | Simpoh gajah | 5 |  |  |  | (B), (Fl) | F | (F) |
| Dilleniaceae | *Tetracera indica* | Mempelas (climber) | 1 |  |  |  |  | F |  |
| Ebenaceae | *Diospyros areolata* | Kayu arang | 6 |  |  |  | F, L, B | F, L, B | F, L, B |
| Ebenaceae | *Diospyros dictyoneura* | Kayu arang | 1 |  |  |  | F, L, B | F, L, B | F, L, B |
| Ebenaceae | *Diospyros diepenhorstii* | Kayu arang | 1 |  |  |  | L | F, L, B | F, L, B |
| Ebenaceae | *Diospyros lanceifolia* | Kayu arang | 9 |  |  |  | F, L, B | F | F, L, B |
| Elaeocarpaceae | *Elaeocarpus petiolatus* | Mendung | 3 |  |  |  | F | F, L |  |
| Elaeocarpaceae | *Elaeocarpus polystachyus* | Mendung | 1 |  |  |  | F | F, L |  |
| Euphorbiaceae | *Aporosa arborea* | Sebasah | 2 |  |  |  | F, L | F, L, B |  |
| Euphorbiaceae | *Aporosa aurea* | Sebasah | 9 |  |  |  | F, L | F, L, B |  |
| Euphorbiaceae | *Aporosa falcifera* | Sebasah | 4 |  |  |  | F, L | F, L, B |  |
| Euphorbiaceae | *Blumeodendron kurzii* | Gaham badak | 4 |  |  |  | (L) | F, L |  |
| Euphorbiaceae | *Breynia coronata* | Hujan panas | 24 |  |  |  |  |  |  |
| Euphorbiaceae | *Drypetes longifolia* | Pelir tikus | 1 |  |  |  | B | F, L, B | F |
| Euphorbiaceae | *Drypetes pendula* | Gelugor salah | 2 |  |  |  |  | F, L, B | F |
| Euphorbiaceae | *Macaranga conifera* | Mahang | 1 |  |  |  |  | F |  |
| Euphorbiaceae | *Macaranga gigantea* | Koben | 10 |  |  |  |  | F, B, Fl |  |
| Euphorbiaceae | *Microdesmis caseariifolia* | - | 1 |  |  |  |  |  |  |
| Fabaceae | *Archidendron microcarpum* | Kekacang | 1 |  |  |  |  | L |  |
| Fabaceae | *Callerya atropurpurea* | Tulang daing | 2 |  |  |  | F |  |  |
| Fabaceae | *Crudia scortechinii* | Kekacang | 1 |  |  | B | (B) | F |  |
| Fabaceae | *Dialium indum* | Keranji | 14 |  |  |  | B | F |  |
| Fabaceae | *Dialium laurinum* | Keranji tebal besar | 1 |  |  |  |  | F, L, B |  |
| Fabaceae | *Koompassia excelsa* | Tualang | - | L |  |  | F | L, B |  |
| Fabaceae | *Ormosia sumatrana* | - | 1 |  |  |  |  | L |  |
| Fabaceae | *Sindora coriacea* | Sepetir | 14 | L |  | L | L |  |  |
| Fagaceae | *Castanopsis inermis* | Berangan | - | F | F | F, L, B | F | F, L, B | (F) |
| Fagaceae | *Lithocarpus encleiscarpus* | Mempening | 2 |  |  |  | F, B | F, L, B, Fl | (F) |
| Flacourtiaceae | *Paropsia vareciformis* | Dendulang | 5 |  |  |  |  |  |  |
| Flacourtiaceae | *Scaphocalyx spathacea* | - | 1 |  |  |  |  |  |  |
| Ixonanthaceae | *Ixonanthes icosandra* | Pagar anak | 45 |  |  | L |  |  |  |
| Lauraceae | *Actinodaphne sp.* | Medang payung | 5 |  |  |  |  | F |  |
| Lauraceae | *Alseodaphne nigrescens* | Medang | 6 |  |  |  |  | F |  |
| Lauraceae | *Belischmiedia sp.* | Medang | 1 |  |  |  | B |  | F |
| Lauraceae | *Cinnamomum porrectum* | Medang sarsi | 4 |  |  |  |  | F, B | (L) |
| Lauraceae | *Cinnamomum zeylanica* | Medang | - | L |  |  |  | F, B | (L) |
| Lauraceae | *Lindera subumbellifera* | Medang | 2 |  |  |  |  | F |  |
| Lauraceae | *Litsea costalis* | Medang daun lebar | 9 |  |  |  | F, L |  |  |
| Lauraceae | *Litsea elliptica* | Medang kesing | 33 |  |  |  | F, L |  |  |
| Lauraceae | *Litsea robusta* | Medang | 1 |  |  |  | F, L | F, L |  |
| Lauraceae | *Litsea sp.* | Medang | 2 |  |  |  | F, L | F, L, B, Fl |  |
| Lauraceae | *Litsea spathacea* | Medang | 2 |  |  |  | F, L |  |  |
| Malvaceae | *Heritiera simplicifolia* | Mengkulang siku keluang | 2 |  |  |  |  |  |  |
| Malvaceae | *Heritiera sumatrana* | Mengkulang | 1 |  |  |  |  |  |  |
| Melastomataceae | *Memecylon dichotomum* | Nipis kulit | 1 |  |  |  |  | F, L, B | F, L |
| Melastomataceae | *Pternandra echinata* | - | 1 |  |  |  |  | F, L | (F) |
| Meliaceae | *Aglaia forbesii* | Bekak | 7 |  |  |  |  | F, L, B | F |
| Meliaceae | *Aglaia tomentosa* | Bekak | 1 |  |  |  | F | F | F |
| Meliaceae | *Aphanamixis polystachya* | Bekak | 1 |  |  |  |  | (F) | (F) |
| Meliaceae | *Chisocheton pauciflorus* | Bekak | 1 |  |  |  | F | (F) |  |
| Moraceae | *Artocarpus integer var. silvestris* | Bangkong | 10 |  |  |  | F, L, B | **(F), (L), (B), (Fl)** | F, L, B |
| Moraceae | *Artocarpus kemando* | Pudu | 7 |  |  |  | F, L, B | F, L, B | F, L, B |
| Moraceae | *Artocarpus lowii* | Miku | 3 |  |  |  | F, L, B | F, L, B | F, L, B |
| Moraceae | *Ficus fistulosa* | Ara | 4 |  |  |  | F, L, B | F, L, B | F, B |
| Myristicaceae | *Horsfiedlia polyspherula* | Penarahan | 1 |  |  |  |  |  | (B) |
| Myristicaceae | *Knema glauca* | Penarahan | 5 |  |  |  | F, Fl | F, L, B | (L), B |
| Myrtaceae | *Rhodamnia cinerea* | Mempoyan | 1 |  |  |  |  | F |  |
| Myrtaceae | *Syzygium attenuatum* | Kelat | 6 |  |  |  | F | F, L, B |  |
| Myrtaceae | *Syzygium cinereum* | Kelat | 36 |  |  |  | F | F, L, B |  |
| Myrtaceae | *Syzygium filiforme* | Kelat | 1 |  |  |  | F | F, L, B |  |
| Myrtaceae | *Syzygium sp.* | Kelat | 1 |  |  |  | F | F, L, B |  |
| Olacaceae | *Ochanostachys amentacea* | Petaling | 12 |  |  |  | F |  |  |
| Olacaceae | *Strombosia javanica* | Dali-dali | 6 |  |  |  |  |  |  |
| Oxalidaceae | *Sarcotheca monophylla* | Asam popoi | 33 |  |  |  |  | F | (F), (L) |
| Phyllanthaceae | *Baccaurea brevipes* | Rambai hutan | 5 | L |  |  |  |  |  |
| Phyllanthaceae | *Baccaurea parviflora* | Setambun | 3 |  |  |  |  | F |  |
| Polygalaceae | *Xanthophyllum affine* | Minyak beruk | 37 |  |  |  | L, Fl | L | F, L |
| Polygalaceae | *Xanthophyllum griffithii ssp. Angustifolium* | Minyak beruk | 1 |  |  |  | F, L | F | F, L |
| Rubiaceae | *Aidia densiflora* | Menterbang | 11 |  |  |  |  |  |  |
| Rubiaceae | *Canthium glabrum* | Sebusuk | 2 |  |  |  |  | F, B | B |
| Rubiaceae | *Psydrax maingayi* | Kemuning | 3 |  |  |  |  | F |  |
| Rubiaceae | *Urophyllum glabrum* | Mata kerling | 2 |  |  |  |  | F |  |
| Ruscaceae | *Dracaena maingayi* | Senjuang hutan | 10 |  |  |  |  | L |  |
| Rutaceae | *Melicope lunu-ankenda* | Tenggek burung | 4 |  |  | L |  |  |  |
| Sapindaceae | *Nephelium cuspidatum var. eriopetalum* | Lotong | 27 |  |  |  | F, L | F | F |
| Sapindaceae | *Nephelium juglandifolium* | Rambutan hutan | 9 |  |  |  | F, L |  | F |
| Sapindaceae | *Nephelium lappaceum var. lappaceum* | Rambutan | 14 |  |  |  | F, L | F, L | F |
| Sapindaceae | *Nephelium ramboutan-ake* | Pulasan | 3 |  |  | L | F | F, L | F |
| Sapotaceae | *Pouteria malaccensis* | Nyatoh | 1 |  |  |  |  | F, L, B, Fl |  |
| Sterculiaceae | *Sterculia cordata* | Mengkulang | 2 |  |  |  |  | F, L, B | (F) |
| Verbenaceae | *Vitex pinnata* | Halban | 6 |  |  |  |  | L |  |
| Violaceae | *Rinorea anguifera* | Pacat gemuk | 2 |  |  |  |  | F |  |
| Vitaceae | *Nothocissus spicifera* | Lakum (climber) | 6 |  |  |  |  |  |  |
|  |  |  | 635 | F1,L5,S1 | F2,L2,S1 | F1,L7,B3 S1,Sh1 |  |  |  |
|  |  |  |  |  |  |  |  |  |  |
|  |  | banana | - | L, S | L, S |  |  |  |  |
|  |  | coconut | - |  | L | L |  |  |  |
